# Ischemic stroke risk during long-term follow up in patients with successful catheter ablation for atrial fibrillation in Korea

**DOI:** 10.1371/journal.pone.0201061

**Published:** 2018-07-19

**Authors:** Dong-Hyeok Kim, Dae-In Lee, Jinhee Ahn, Kwang-No Lee, Seung-Young Roh, Jaemin Shim, Jong-Il Choi, Young-Hoon Kim

**Affiliations:** 1 Division of Cardiology, Department of Internal Medicine, Korea University College of Medicine, Korea University Medical Center, Seoul, Republic of Korea; 2 Division of Cardiology, Chungbuk National University Hospital, Cheongju, Korea; 3 Division of Cardiology, Pusan National University Hospital, Busan, Korea; 4 Division of Cardiology, Dongguk University College of Medicine and Dongguk University Medical Center, Goyang, Korea; University of Palermo, ITALY

## Abstract

The interruption of oral anticoagulation therapy (OAC) after CA of atrial fibrillation (AF) is controversial. The purpose of this study was to evaluate the relationship between successful long-term outcomes of catheter resection and SR maintenance and ischemic stroke risk in Korea. We studied 1,548 consecutive patients who were followed up for more than 2 years after CA of AF. We investigated the incidence of ischemic stroke during long-term follow-up. Compared to the AF recurrence group (n = 619), the sinus rhythm (SR) maintenance group (n = 929) had more paroxysmal AF (74.6% versus 44.4%, p<0.001), smaller LA size (39.9±5.7mm versus 42.3±6.0mm, p<0.001), and younger age (54.2±10.9 years versus 56.4±10.6 years, p<0.001). However, CHA_2_DS_2_-VASc scores were not significantly different between the two groups (0.9 vs. 1.1, p = 0.053). The overall incidence of ischemic stroke during the mean follow-up period of 54 months after CA was 0.6%, and was significantly lower in the SR group than the AF recurrence group (0.3% vs. 1.1%, log-rank test p<0.001). However, in sub-analysis in the SR group, the rate of ischemic stroke was significantly increasing in patients with a CHA_2_DS_2_-VASc score ≥ 4 compared to those with a CHA_2_DS_2_-VASc score < 4 (4.3% vs. 0.2%, log-rank test p<0.001). In conclusion, this long-term follow-up data in patients with AF who underwent successful CA showed that SR maintenance was correlated with a lower rate of ischemic stroke in Korea. However, it was only observed in patients with CHA_2_DS_2_-VASc score ≤3.

## Introduction

Current guidelines demonstrate that atrial fibrillation (AF) catheter ablation (CA) to restore sinus rhythm (SR) should not be performed only for stop of anticoagulation, which is a class III recommendation with level C evidence [1]. The CHA_2_DS_2_-VASc score is the only risk stratification tool for oral anticoagulation therapy (OAC) before and after CA [2]. This recommendation is based on the belief that the baseline risk of thromboembolic events (TE) remains unchanged despite successful CA [3]. However, it is reasonable to speculate that elimination of AF may reduce the risk of TE.

Many studies have demonstrated that OAC can be discontinued after successful CA for patients with a relatively low risk of TE events [4–6]. Saad EB et al. showed that there is no significant TE-related morbidity of patients with antiarrhythmic drugs (AAD) and discontinuation of OAC after successful CA and CHADS_2_ scores ≤3 [4]. Roger A. A nationwide cohort study in Denmark also demonstrated that TE risk beyond 3 months after CA was relatively low compared with a matched non-ablated AF cohort [5]. A study from the Swedish health registries showed that ablation may be associated with a lower incidence of ischemic stroke and death in patients with AF [6]. However, this is not clear in Korea patients. Therefore, we hypothesized that patients after successful CA experience fewer TE events if there is a long-term SR maintenance and a relatively low stroke risk in Korea.

## Methods

### Study population

**Fig 1:**
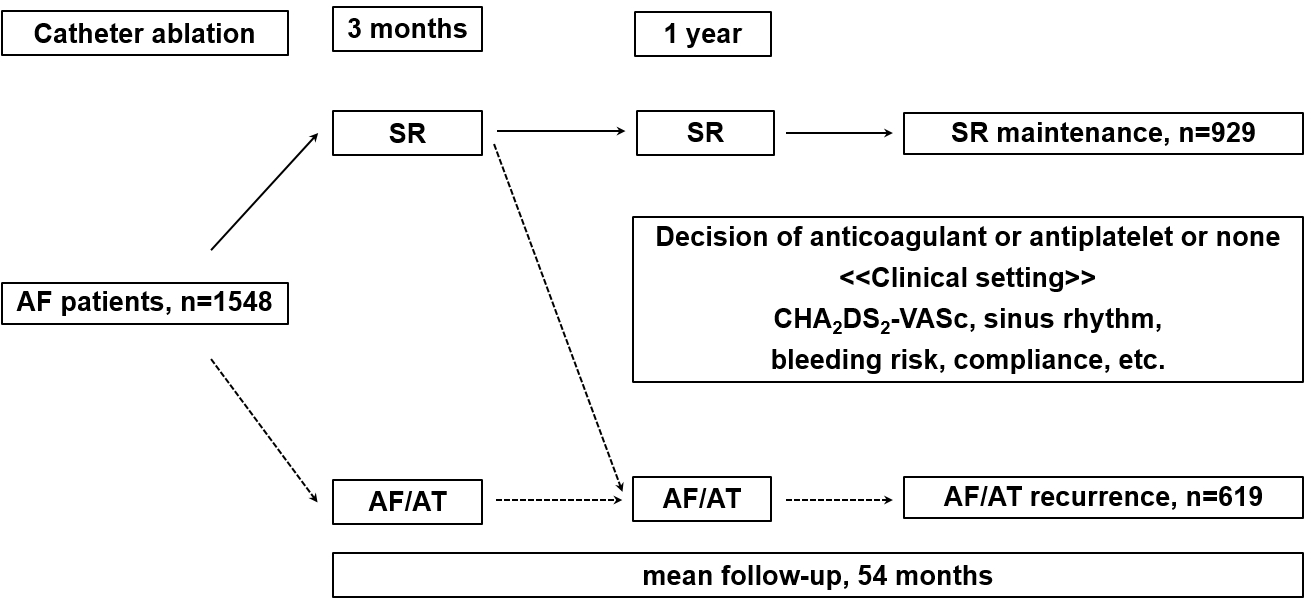
Study flow chart and patient population. The flow diagram shows patients population, AF recurrence and sinus rhythm maintenance group after 1 year RFCA.

We studied 1,548 consecutive patients with AF and more than 2 years of follow-up from February 2000 to March 2013. Total 1,548 patients were divided into two groups (Fig 1). The SR maintenance group was defined as patients who underwent CA and remained in SR even after 1 year. The recurrence group was defined as patients who underwent CA and had an AF recurrence within 1 year. If a patient who had AF recurrence after 1 year, the patient was defined as SR maintenance group because that AF was not documented and SR maintenance until 1 year. This study was approved by the institutional review board in Korea University Medical Center (AN17210-002). Consent was waived by the ethics committee.

### Procedures for catheter ablation

Prior to the CA procedure, all antiarrhythmic drugs were discontinued, and more than 5 half-lives were allowed to pass. Circumferentially antral pulmonary vein isolation (PVI) with electrical isolation was performed. When AF followed PVI, either linear ablation or complex fractionated electrogram (CFAE) ablation was also performed. The endpoints of the ablation were AF or AT termination. During the enrollment period, the CA strategy was changed from PVI in paroxysmal AF to combination of PVI with linear ablation or/and CFAE ablation in persistent or long-standing persistent AF.

### Follow-up

To assess the efficacy of catheter ablation, we investigated freedom from atrial tachyarrhythmia (= AF or AT) after the procedure. After ablation, patients were asked to visit the outpatient clinic at 1, 3, 6, and 12 months and then every 6 months thereafter or whenever they experienced tachycardia-related symptoms. Electrocardiogram (ECG) was performed at every visit. Holter monitor recording was performed in patients who were thought to have arrhythmia-related intermittent symptoms. Recurrence of atrial tachyarrhythmia was defined as an event lasting more than 30 seconds after a 3-month blanking period. AADs were taken during the first 3 months after the ablation. Discontinuation of AADs was determined at the physicians’ discretion. During follow-up, ischemic stroke was investigated by a neurologist’s diagnosis and brain imaging.

### Statistical analyses

All values are expressed as means ± SD or as numbers and percentages where appropriate. Categorical data were compared with the χ^2^ test. Continuous variable data were compared by the independent samples t-test when the distribution was normal or by the Mann-Whitney U test if it the distribution was not normal. Kaplan-Meier analysis with the log-rank test was used to determine the probability of ischemic stroke. P<0.05 was considered statistically significant. Statistical analyses were performed using SPSS Statistics 19.0 software (SPSS Inc., Chicago, IL, USA).

## Results

### Clinical characteristics

Clinical characteristics at baseline are summarized in Table 1. Compared to the AF recurrence group (n = 619), the SR maintenance group (n = 929) had younger age (54.2±10.9 years versus 56.4±10.6 years, p<0.001), shorter time of AF onset (31.0±43.9 months versus 44.9±58.7 months, p<0.001), more paroxysmal AF (74.6% versus 44.4%, p<0.001), and smaller LA size (39.9±5.7mm versus 42.3±6.0mm, p<0.001). However, CHA_2_DS_2_-VASc scores were not significantly different between the two groups (0.9 vs. 1.1, p = 0.053).

**Table 1:** Clinical baseline characteristics of the study participants who underwent catheter ablation.

	Total (n = 1548)	AF recurrence (n = 619)	SR maintenance (n = 929)	*p* value
Age, year-old	55.0±10.8	56.4±10.6	54.2±10.9	<0.001
Male sex, n (%)	1228 (79.3)	492 (79.5)	736 (79.2)	0.949
AF onset, months	36.5±50.8	44.9±58.7	31.0±43.9	<0.001
Paroxysmal AF, n (%)	968 (62.6)	275 (44.4)	693 (74.6)	<0.001
LVEF, %	55.2±6.0	54.6±6.4	55.7±5.7	<0.001
LA size, mm	40.8±5.9	42.3±6.0	39.9±5.7	<0.001
CHF, n (%)	50 (3.2)	24 (3.9)	26 (2.8)	0.244
Hypertension, n (%)	482 (31.1)	217 (35.1)	265 (28.5)	0.007
Diabetes mellitus, n (%)	109 (7.0)	53 (8.6)	56 (6.0)	0.068
CHAS_2_DS_2_-VASc score	1.0±1.1	1.1±1.1	0.9±1.1	0.053
0	667 (43.0)	228 (36.8)	439 (47.3)	<0.001
1	492 (31.7)	205 (33.1)	287 (30.9)	0.193
≥2	389 (25.1)	186 (30.0)	203 (21.8)	<0.001

Values are expressed as means ± SDs and numbers (percentages). AF; atrial fibrillation, LVEF; left ventricular ejection fraction, LA; left atrium, CHF; congestive heart failure

### AAD use and antithrombotic therapy after CA

Table 2 shows AAD use and antithrombotic therapy after CA. After CA, AAD use was more frequent in the AF recurrence group compared to the SR maintenance group (64.0% versus 44.6%, p<0.001). After CA, antithrombotic therapy was more frequent in the AF recurrence group compared to the SR maintenance group (88.7% versus 72.2%, p<0.001). The SR group had more antiplatelet therapy than the AF recurrence group (58.8% versus 53.5%, p = 0.036). However, OAC was more frequent in the AF recurrence group compared to the SR maintenance group (36.5% versus 14.3%, p<0.001). Interestingly, in the SR group, the rate of OAC for patients with a CHA_2_DS_2_-VASc score ≥2 was only 16.7% and the rate of antiplatelet therapy was 70.9%.

**Table 2:** Antiarrhythmic drug and antithrombotic therapy after catheter ablation between the AF recurrence and SR maintenance groups.

	Total (n = 1548)	AF recurrence (n = 619)	SR maintenance (n = 929)	*p* value
AAD†	810 (52.3)	396 (64.0)	414 (44.6)	<0.001
Antithrombotic therapy‡	1220 (78.8)	549 (88.7)	671 (72.2)	<0.001
Antiplatelet therapy‡	876 (56.5)	330 (53.5)	546 (58.8)	0.036
Anticoagulation therapy‡	359 (23.1)	226 (36.5)	133 (14.3)	<0.001

Values are expressed as numbers (percentages). AAD; antiarrhythmic drug

† 3 months after catheter ablation

‡ 1 year after catheter ablation

### Ischemic stroke events after successful CA

**Fig 2:**
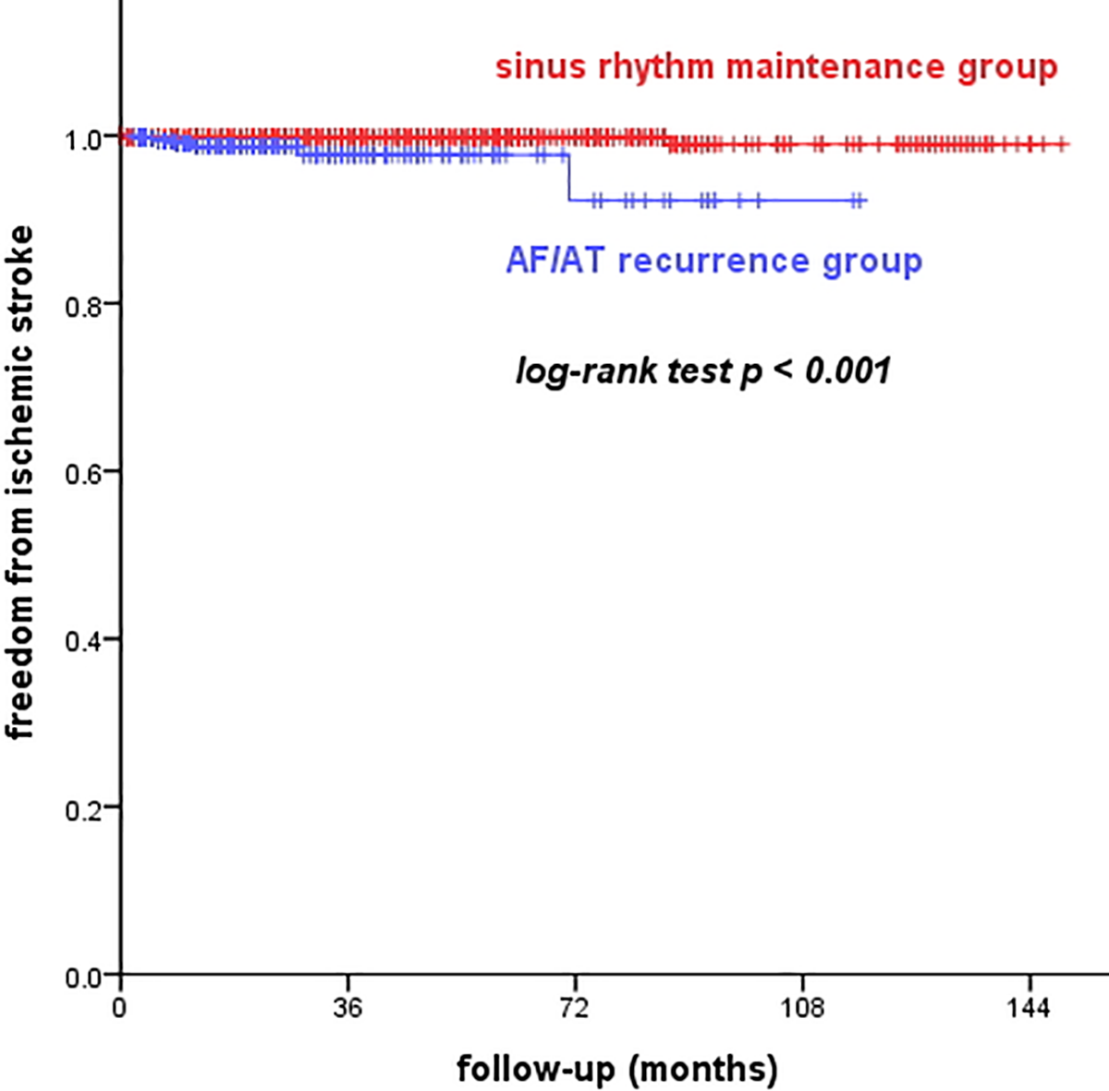
Comparison of freedom from atrial tachyarrhythmia after catheter ablation. The Kaplan-Meier survival curves for freedom from ischemic stroke after successful RFCA between AF recurrence and sinus rhythm maintenance group.

The overall incidence of ischemic stroke after CA was 0.6% in the follow-up period of 54 months. The incidence of ischemic stroke was significantly lower in the SR maintenance group than in the AF recurrence group (0.3% vs. 1.1%, log-rank test, p<0.001, Fig 2).

#### 1) CHA_2_DS_2_-VASc score

Ischemic stroke events are shown in Table 3. In the SR maintenance group, most ischemic events were reported in patients with high CHA_2_DS_2_-VASc scores: CHA_2_DS_2_-VASc score 0 (n = 1, 0.2%), CHA_2_DS_2_-VASc score 4 (n = 1, 5.5%), CHA_2_DS_2_-VASc score 5 (n = 1, 25%). However, in the AF recurrence group, ischemic stroke events were reported in patients with low and high CHA_2_DS_2_-VASc scores: CHA_2_DS_2_-VASc score 0 (n = 2, 0.9%), CHA_2_DS_2_-VASc score 1 (n = 4, 1.9%), CHA_2_DS_2_-VASc score 5 (n = 1, 11.1%). All 10 patients with stroke events were on OAC in 3 in the SR group and 7 in the recurrence group. There was no difference of OAC therapy for patients with stroke events after ablation in both groups.

**Table 3:** Distribution of ischemic stroke events between the AF recurrence and SR maintenance groups.

CHA_2_DS_2_-VASc	Total (n = 1548)		AF recurrence (n = 619)		SR maintenance (n = 929)		*p* value†
	Patients	Events	Patients	Events	Patients	Events	
0	667	3 (0.4)	228	2 (0.9)	439	1 (0.2)	
1	492	4 (0.8)	205	4 (1.9)	287	0	
2	228	0	113	0	115	0	
3	110	0	45	0	65	0	
4	36	1 (2.7)	18	0	18	1 (5.5)	
5	13	2 (15.3)	9	1 (11.1)	4	1 (25.0)	
6	2	0	1	0	1	0	
≥7	0	0	0	0	0	0	
Total	1548	10 (0.6)	619	7 (1.1)	929	3 (0.3)	<0.001

Values are expressed as numbers (percentages).

† *p* value: compared AF recurrence versus SR maintenance

#### 2) Risk factors for ischemic stroke

Table 4 shows risk factors for ischemic stroke. Univariate analysis showed lower CHA_2_DS_2_-VASc score (HR 1.540, p = 0.049), AAD use after CA (HR 0.207, p = 0.048), and SR maintenance (HR 0.122, p = 0.005) reduced the risk of ischemic stroke. However, multivariate analysis showed SR maintenance (HR 0.151, p = 0.013) was the only factor that reduced risk.

**Table 4:** Cox regression analysis for stroke risk.

Factors	Hazard ratio (95% CI) univariate analysis	P value	Hazard ratio (95% CI) multivariate analysis	P value
Age, year-old	1.06 (0.99–1.13)	0.055		
Male sex, n (%)	0.364 (0.10–1.29)	0.364		
Previous stroke	1.825 (0.64–5.15)	0.256		
CHF	21.2 (0.00–7.53)	0.691		
Vascular diseases	0.295 (0.03–2.33)	0.248		
AF type, paroxysmal	0.303 (0.08–1.09)	0.068		
CHAS_2_DS_2_-VASc	1.540 (1.00–2.36)	0.049	1.421 (0.92–2.18)	0.109
AAD use after CA	0.207 (0.04–0.98)	0.048	0.269 (0.05–1.31)	0.105
OAC after CA	0.001 (0.00–3.77)	0.327		
SR versus recurrence	0.122 (0.02–0.52)	0.005	0.151 (0.03–0.67)	0.013

Values are expressed as HR (95% CI). CI; confidence interval, CHF; congestive heart failure, AAD; antiarrhythmic drug, CA; catheter ablation, OAC; oral anticoagulation therapy, SR; sinus rhythm

### Cut off-value of CHA_2_DS_2_-VASc score for ischemic stroke after CA

The CHA_2_DS_2_-VASc score is already the main tool for stratification of ischemic stroke risk. Table 5 shows Cox regression analysis for stroke risk in the SR maintenance group. CHA_2_DS_2_-VASc score was the main factor for stroke risk (HR 2.11, CI 1.08–4.12, p = 0.028). However, the cut-off value for differentiating stroke events was not CHA_2_DS_2_-VASc score ≥2 (HR 0.11, CI 0.01–1.21, p = 0.072) nor ≥3 (HR 0.16, CI 0.01–1.89, p = 0.149), but ≥4 (HR 17.65, CI 1.59–195.5, p = 0.019). In patients with CHA_2_DS_2_-VASc score of 4 or more, the risk of ischemic stroke increased even after successful CA and SR maintenance. However, this showed the trend of low stroke event in CHA2DS2-VAS 2 or 3 in patients with SR maintenance after successful ablation.

**Table 5:** Cox regression analysis for stroke risk in sinus rhythm.

Factors	Hazard ratio (95% CI) univariate analysis	P value
Age, year-old	1.12 (0.99–1.26)	0.067
Male sex, n (%)	2.16 (0.19–24.2)	0.529
Previous stroke	20.98 (0.00–20.98)	0.849
CHF	21.30 (0.00–1.52)	0.826
Vascular diseases	0.06 (0.01–0.67)	0.023
AF type, paroxysmal	30.71 (0.00–2.78)	0.556
CHAS_2_DS_2_-VASc	2.11 (1.08–4.12)	0.028
AAD use after CA	0.01 (0.00–144.3)	0.339
OAC after CA	0.01 (0.00–5.70)	0.519
CHAS_2_DS_2_-VASc ≥2	0.11 (0.01–1.21)	0.072
CHAS_2_DS_2_-VASc ≥3	0.16 (0.01–1.89)	0.149
CHAS_2_DS_2_-VASc ≥4	17.65 (1.59–195.5)	0.019

Values are expressed as HR (95% CI). CI; confidence interval, CHF; congestive heart failure, AAD; antiarrhythmic drug, CA; catheter ablation, OAC; oral anticoagulation therapy, SR; sinus rhythm

## Discussion

### Main findings

This long-term follow-up data in patients with AF who underwent catheter ablation showed that SR maintenance after successful CA was correlated with a lower rate of ischemic stroke. However, it was only observed in patients with CHA_2_DS_2_-VASc score ≤3 in Korea.

### Comparison of previous studies of ischemic stroke after catheter ablation

Many studies demonstrated that OAC can be discontinued after successful CA for patients with a relatively low risk of TE [4–9]. According to the current guidelines [1], even after CA of AF, anticoagulation therapy should be continued when the CHA_2_DS_2_-VASc score remains ≥2. This recommendation is based on the belief that the baseline risk of TE remains unchanged despite a successful CA [3]. However, there is no randomized study to support this, and this guideline recommendation is class III, but evidence level C. A study from a Danish cohort showed that TE risk beyond 3 months after CA was relatively low compared with a matched non-ablated AF cohort [5]. The bleeding risk score HAS-BLED increased with CHA_2_DS_2_-VASc score. Practical clinicians take bleeding risk into consideration. Karasoy D et al. also emphasized that serious bleeding risk associated with OAC seems to outweigh the benefits of TE risk reduction [5]. When considering the SR maintenance duration, another study showed that the risk of stroke is low in patients with no recurrence in first 1 year after CA [7]. In our study, we defined patients with no AF recurrence for at least 1 year as the SR maintenance group, and this was consistent with their results [7].

Another study pointed out a CHA_2_DS_2_-VASc cut-off value for stroke [4]. Saad EB et al. demonstrated that no significant TE-related morbidity was observed when AAD and OAC were discontinued after successful CA in patients with a CHADS_2_ score ≤3 who were maintained on antiplatelet therapy during long-term follow-up. This suggests the existence of a gray zone which has a relatively low risk of ischemic stroke after successful CA. In our results, antiplatelet therapy was used more than OAC in the SR group than the recurrence group (70.9% versus 16.7%, p<0.001, respectively). CHA_2_DS_2_-VASc scores of 2 and 3 are relatively low-risk if SR is maintained after successful CA. Themistoclakis S et al. showed that the risk-benefit ratio favored the suspension of OAC after successful CA in patients at moderate-high risk of TE [10]. They also emphasized that the CHADS_2_ score system probably is not the most appropriate system for assessing TE risk and establishing an anticoagulation strategy after CA.

On contrary, there were studies which concluded inevitable OAC after successful CA [11–15]. Oral H et al. demonstrated that sufficient safety data are as yet unavailable to support discontinuation of OAC in patients older than 65 years or with a history of stroke [11]. Patients older than 65 years or with a history of stroke have a high risk of TE events and higher CHA_2_DS_2_-VASc scores. If those patients have a history of stroke and older than 65 years, CHA_2_DS_2_-VASc scores is 3 in male and 4 in female. Our results showed that stroke events mostly occurred in patients with CHA_2_DS_2_-VASc scores ≥ 4, and the cut-off value for differentiating ischemic stroke events was ≤3. The number of stroke event was very low in SR group. And stroke event was mostly detected in patients CHA2DS2-VASc score ≥4 (Table 3). Therefore, this showed only trend of distribution of stroke event and further large study would be needed. The ESS-PRAFA study also showed that after CA, most patients (89.3%) continued the same anticoagulant as before CA [15]. This trend toward practical OAC was based on the CHA_2_DS_2_-VASc score, but successful CA rate and rhythm status. However, in our results, rhythm status was the most significant independent predictor of ischemic stroke.

In AFFIRM study, Corley SD et al. showed that rhythm control is not superior to rate control [16]. However, they demonstrated that OAC with warfarin improved survival, but SR was an important determinant of survival. The rhythm control strategy with CA improved more favorable outcomes than the rhythm control strategy with AAD alone or a rate control strategy [17–20]. CA has been improved and is an efficient tool for maintaining SR [21]. If TE are assessed not only by CHA_2_DS_2_-VASc score but also SR maintenance, modified OAC may be required after successful CA. However, our study showed that it was only observed in patients with CHA_2_DS_2_-VASc score ≤3. We cannot recommend discontinuation of OAC for patient with SR maintenance after ablation. However, this study shows and emphasizes that gray zone of stroke risk exists. There was a low risk of stroke in patients with SR maintenance and CHA2DS2-VASc 2 or 3 after successful ablation. Further randomized and large study should be needed for recommendation of discontinuation.

## Study limitations

First, this was not a randomized trial, but a retrospective study. However, over the past 10 years, the CA strategy has developed from PVI to linear or/and CFAE ablation, the rate of successful CA has improved, the ratio of sinus rhythm maintenance is high, and the rate of total ischemic stroke events remains low. Therefore, there were low rates of OAC for patients with CHA_2_DS_2_-VASc scores ≥2, based on rhythm status, bleeding risk, and compliance. Second, there is no analysis of asymptomatic AF episodes which were not detected by ECG or Holter monitoring. Like other AF studies, asymptomatic AF episodes are important and limit the analysis of AF recurrence. This would underestimate the recurrence of AF after ablation, and result in misallocation of some patients from recurrence group to SR group. Third, the study population included patients with a mean CHA_2_DS_2_-VASc score of 1.0 and a small number with a moderate risk of TE events. Therefore, 0.6% of the absolute ischemic stroke event rate was very low. Patients with CHA_2_DS_2_-VASc scores 0 or 1 would not be recommended OAC by current guidelines, regardless of rhythm status or success of ablation. CHA_2_DS_2_-VASc scores were not significantly different between the two groups (0.9 vs. 1.1, p = 0.053). Comparison and p value of CHA2DS2-VASc score = 0, 1, and ≥2 were <0.001, 0.193, <0.001, respectively. Patients with CHA2DS2-VASc score ≥2 were lower in SR group. This finding was also limitation of interpretation of stroke events in both groups. Large population studies with higher CHA_2_DS_2_-VASc scores are necessary to evaluate the role of sinus rhythm status after successful CA to further address our question. Finally, the ratio of male is much higher than that of female both AF recurrence (79.5%) and SR maintenance group (79.2%). This had the limitation of stroke risk for female population. Mean LVEF showed normal function both AF recurrence (54.6%) and SR maintenance group (55.7%). This also had the limitation of evaluating catheter ablation for atrial fibrillation in patients with reduced LVEF. And further study would be needed.

## Conclusions

This long-term follow up study in patients with AF who underwent catheter ablation showed that sinus rhythm maintenance was correlated with a lower rate of ischemic stroke in Korea. However, it was only observed in patients with CHA_2_DS_2_-VASc score ≤3.

## Abbreviations

AF: atrial fibrillation
CA: catheter ablation
SR: sinus rhythm
OAC: oral anticoagulation therapy
TE: thromboembolic events
AAD: antiarrhythmic drugs
PVI: pulmonary vein isolation
CFAE: complex fractionated electrogram
ECG: electrocardiogram
